# Insights from national stakeholders and health workers on learning and performance interventions in immunisation programs: a multi-country situational analysis

**DOI:** 10.7189/jogh.15.04109

**Published:** 2025-03-28

**Authors:** Julia Bluestone, Emily Bryce, Alexander K Rowe, Naina J Ahuja, Wincate M Murathi, Rosemary N Njogu, Arshad Chandio

**Affiliations:** 1 Program and Technical Excellence Office, Jhpiego, Baltimore, Maryland, USA; 2 Monitoring, Evaluation and Research, Jhpiego, Baltimore, Maryland, USA; 3 Independent Contractor, Health Systems and Immunisation Strengthening team, Gavi, the Vaccine Alliance, Geneva, Switzerland; 4 Health Programme Group, Maternal, Newborn, Child, and Adolescent Health Section, Unit for Digital Health and Information Systems, United Nations Children’s Fund, New York, New York, USA; 5 Program Team, Jhpiego, Nairobi, Kenya; 6 Technical Maternal, Newborn, and Child Health Team, Jhpiego, Nairobi, Kenya; 7 Consultant, Jhpiego, Islamabad, Pakistan

## Abstract

**Background:**

Health workers play a key role in providing high-quality health services, but health worker practice improvements remain limited despite significant investments in learning and performance interventions. We conducted a situational analysis to explore factors affecting health worker performance, focusing on barriers and facilitators and integrating digital solutions.

**Methods:**

In the analysis we focussed on paid professional health workers. Primary data collection occurred between April–May 2022 across seven countries, involving key informant interviews with immunisation program managers and human resource representatives. In three countries, human-centred design meetings included surveys on preferred interventions for improving learning and performance. Secondary data included a desk review of the literature, including recent strategy documents from the Gavi Alliance. We used a virtual session with human-centred design facilitators to create a health worker learning journey map.

**Results:**

Our findings show a shift towards digital and innovative approaches in learning, though traditional methods, such as in-service training and supervision, still dominate. Most initiatives depend on donor funding. There is a lack of evidence on the effectiveness of digital solutions. Integration with health workers’ continuing professional development processes is limited, but career advancement motivates engagement. Challenges include inadequate staffing, limited training opportunities, and poor digital infrastructure. Preferred methods include workplace-based learning and digitally supported training. Evidence supports quality improvement or group problem-solving to improve practices, while other approaches, such as eLearning or blended learning and mentorship, require further evaluation.

**Conclusions:**

Stakeholders, including donors, should prioritise support for more effective learning approaches, combining strategies to improve outcomes. While stakeholders desire to expand digital learning, given the limited evidence, prioritising effectiveness evaluations are crucial. Educating stakeholders on evidence-based practices, promoting combined strategies, evaluating unproven interventions, and aligning donor funding with effective approaches is critical to enhancing interventions.

Health workers (HWs) are essential for providing high-quality health services. However, health systems challenges, such as insufficient staffing, deficient infrastructure, and shortages of supplies and commodities, are significant constraints on HWs and quality of care [1,2]. There is a growing recognition that stand-alone, classroom-based, in-service training and traditional supervision alone are not sufficient to improve HWs performance [3]. However, there is a wide variety of other interventions to support improved HWs learning and performance, some of which are more effective than training and supervision at influencing HWs practices [4].

The shortage of HWs remains a critical barrier to improving health outcomes in low- and middle-income countries (LMICs) [3]. LMICs face acute health workforce shortages and inequitable distribution of skilled HWs, which impact delivering essential health services. During the COVID-19 pandemic, there was an explosion of digital solutions used to replace in-person training. Given the shortage and burden on HWs, the need to expand access to learning and performance improvement opportunities for HWs, and the rapid increase in the use of digital solutions, there is a need to better understand the evidence and potential role of digital solutions in addressing HWs learning and performance needs. To ensure clarity of terms, we developed a glossary of definitions for learning and performance interventions and digital education interventions (Table S1–2 in the **Online Supplementary Document****)**. Some of these terms and approaches overlap and are not mutually exclusive.

Gavi, the Vaccine Alliance is comprised of core partners, World Health Organization (WHO), the United Nations Children’s Fund (UNICEF), the World Bank, the United States Centers for Disease Control and Prevention (US CDC) – and other partners such as civil society, the private sector, and other United Nations agencies. The alliance’s goal is to save lives by increasing vaccine use. To improve the impact of investments in human resources for health [5], Gavi, with Rockefeller Foundation support, contracted Jhpiego to complete a situational analysis of HW learning and performance investments, focusing on stakeholder insights and the potential for digital solutions to improve access to learning for those in immunisation programs. The voices of country stakeholders and frontline HWs were an essential component of this analysis because much of the previously published literature lacks the perspectives of country stakeholders on learning and performance interventions.

This situational analysis contributes to understanding priority interventions to improve HW learning and performance and stakeholder insights on future investments in HW learning and performance. It highlights the need to understand the typical HW's learning journey post-graduation and the barriers they face when engaging in learning and performance efforts. The focus of this situational analysis was not specific to challenges around immunisation as most HWs involved in immunisation are not dedicated solely to that task.

## METHODS

### Scope of situational analysis

In the situational analysis, we focussed on post-graduate, paid, professional frontline HWs (*e.g.* nurses, midwives, clinical officers) involved in, but not limited to, immunisation, given HWs’ broader responsibilities. It excluded Community HWs (CHWs) and volunteers. We chose this audience because they constitute the largest proportion of HWs actively engaged in immunisation service delivery, and the legal and regulatory frameworks governing re-licensure and ongoing continued professional development (CPD) requirements currently apply to professional HWs but not to CHWs.

### Procedures and participants

Data collection occurred between April–May 2022 in seven Gavi-supported countries – Pakistan, Kenya, South Sudan, Burkina Faso, Ghana, Tanzania, and Zambia, chosen for their geographic diversity and varying levels of digital and immunisation progress.

The level of engagement in the situational analysis differed by country. In ‘deep-dive’ countries (Pakistan, Kenya, and South Sudan), it included two key informant interviews (KII) with the Expanded Program of Immunisation (EPI) manager and another national immunisation learning and performance management stakeholder. Gavi staff helped identify participants for these interviews to ensure that the participants were involved with and knowledgeable of the landscape. Additionally, in each deep-dive country, a human-centred design (HCD) meeting was conducted with frontline HWs to map their learning and performance journey and understand facilitators and barriers along the pathway. In the four other countries (*i.e.* the non-deep-dive countries), one KII was conducted with the EPI manager for that country. This resulted in a total of 10 KIIs.

The KIIs were either in person at the participant’s place of work or virtual, depending on participant availability and preference. Topics covered familiarity with learning and performance interventions, current practices, funding, barriers and facilitators, and visions for future private-sector and digital solution use. We conducted interviews using a semi-structured interview guide and a companion note-taking template.

The HCD meetings were one-day, in-person sessions with 19–25 HWs each, involving 59 participants across the three countries. We identified and recruited HWs from Jhpiego-supported primary health care facilities. The sample included nurses, midwives, and other mid-level cadres involved in immunisation. Using a collaborative approach, participants developed learner personas and mapped out a typical learning journey from graduation to practice, identifying barriers and facilitators focusing on engaging digital solutions. Participants also completed a survey on learning and performance improvement preferences, using a Likert scale (one – strongly disagree, five – strongly agree) for three categories – learning interventions, digital modalities, and performance management approaches. Participants ranked their preferences within each category, no ‘other’ option was provided.

### Desk review

Secondary data collection involved a desk review limited to published systematic reviews, meta-analyses, and strategic documents on improving HW learning and performance or digital solution integration, shared by the Gavi-convened Health Worker Performance Improvement Technical Working Group (TWG). The desk review began with TWG-recommended systematic reviews and used a snowball search to find recent reviews (2010 and later) in English on PubMed and Google Scholar. It was not meant to be exhaustive, aiming to identify recent key meta-analyses and reviews, excluding those focussed only on CHWs.

### Analysis

We extracted data from national stakeholders’ KII recordings and notes using a template. We summarised data using inductive content analysis to identify commonly reported facilitators, barriers, and preferences for different learning and performance interventions and their implementation. A similar process was applied to the literature review, which linked the extracted data and summaries to objectives regarding effective interventions, barriers, facilitators, and motivators for HW learning and performance. Each deep-dive country produced a learning journey map by reviewing all the assets made during the HCD meetings and debriefing with facilitators. We summarised HWs’ structured feedback and ranking of reported preferences on approaches using descriptive statistics, specifically mean score by country, in Excel (Microsoft, Redmond, Washington, USA).

### Ethical review

The Johns Hopkins University Institutional Review Board reviewed and approved this study (approval number 20667). We obtained informed consent orally from those interviewed prior to their participation in the HCD meetings.

## RESULTS

There are various interventions to improve HW learning and performance. A robust measure of improved performance is improved practice outcomes (*e.g.* increased adherence to treatment guidelines). However, from the desk review, learning outcomes (improved knowledge and/or skills), rather than impact on HW performance (compliance to guidelines, quality care), are measured more often.

### Desk review: intervention effectiveness

We reviewed 39 documents (systematic literature reviews, meta-analyses, and influential reports). A key document included in the desk review was a systematic review of 37 studies conducted by Arsenault et al. that examined intervention effectiveness (in terms of HW practices) over time; these interventions included group problem-solving (also called quality improvement), in-service training, supportive supervision, quality improvement plus training, and in-service training plus supportive supervision. The authors chose these five interventions because they were implemented in facilities (not communities) and there were at least four studies per intervention to conduct the analysis [4]. The primary outcome was the change in effect size over time. Effect size was measured as changes in practice outcomes, such as improved compliance with guidelines, accurate data documentation, and so on. The analysis revealed that, over time, there were reductions in the effectiveness of in-service training alone and training plus supervision, compared to group problem-solving alone, which saw an increase in effectiveness over time. There was no evidence of changes in effectiveness over time for supervision alone. Effect sizes were large immediately after the implementation of group problem-solving plus training, but the analysis found that change over time was not statistically significant [4].

Another systematic review assessed the effectiveness of approaches, irrespective of time. The combination of group problem-solving plus training was found to have the greatest effect size (median (MD) improvement of 56 percentage points (pp)) estimated from four study comparisons, compared to traditional in-service training, which had a small effect size (MD improvement of 10 pp from 78 study comparisons) [6]. Training that includes clinical practice and occurs in the workplace was reported to be more effective than training without these attributes [7].

There is conflicting evidence on training delivered using only digital solutions or platforms to deliver content. Some approaches demonstrated equal or better knowledge outcomes compared to live instruction [8,9], others did not demonstrate knowledge outcome improvements [10], and others showed that digital platforms such as short message service messages sent to HW phones had an effect ranging from zero impact on practice behaviours to a relatively large 24 pp improvement, depending on the study context [11]. WHO advises that the application of digital education, like any other intervention, undergo critical and rigorous evaluation [8].

Additional recommendations to increase the effectiveness of interventions identified from the literature review include combining interventions for greater effect size, in particular, linking quality improvement or group problem-solving efforts to the provision of skill-based training [7] and linking supervision to quality improvement or problem-solving with HWs. Supervision tended to be more effective when supervisors also receive supervision [4]. Additionally, digital solutions that provide access to real-time performance data can make it easier for supervisors to view data in real-time and automate analysis [12].

Findings on the impact of pay-for-performance interventions are conflicting. Financial incentives for performance are often implemented with other interventions; there are few studies of financial incentives in isolation [13]. The literature does caution that pay for performance can have unintended or negative consequences and should be designed carefully [13].

### HCD meetings: a typical HW learning and performance journey

**Figure 1:**
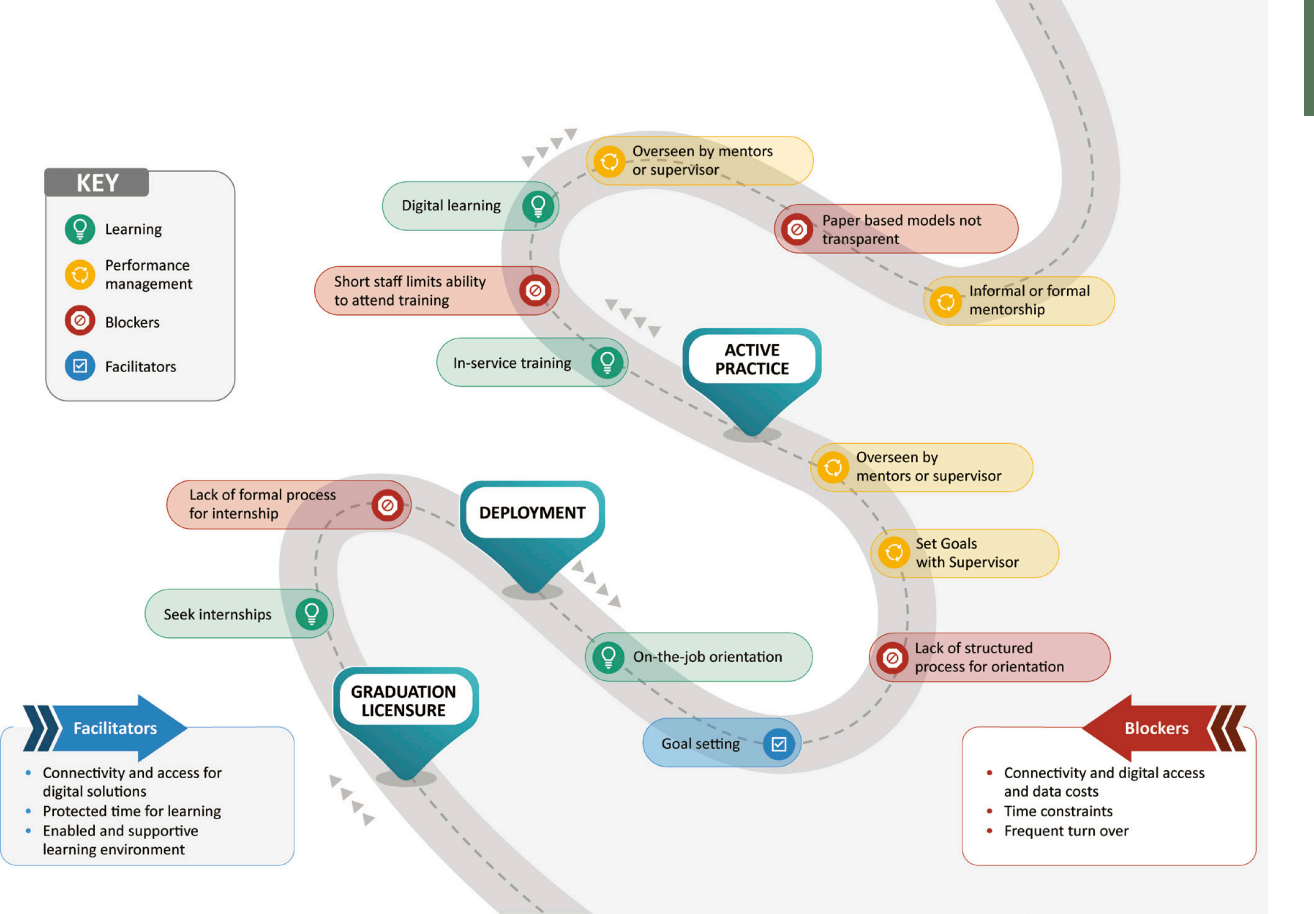
Illustrative learning and performance journey.

For the typical mid-level professional HW, the learning journey has three phases: licensure/post-graduation, deployment, and active practice, which may include re-licensure every two to three years. We used input compiled from the three country HCD meetings to map out the typical learning and performance journey, which depicts key support activities, organised in the order HWs reported them typically occurring (**Figure 1**). The journey map includes the common barriers and facilitators along the learning and performance journey as identified by HWs.

Delays between graduation and deployment can result in a loss of competency. Once deployed, participants often reported either not receiving an orientation or lacking a structured one, making it difficult to understand performance expectations.

During deployment, learning opportunities are often limited, especially in rural or small facilities that are understaffed and cannot release staff for training. Participants noted that training selection is often based on seniority rather than learning needs. Mentorship was frequently mentioned as a key support strategy during deployment, with tools like WhatsApp used to connect with mentors or supervisors. Of the three deep-dive countries – Pakistan, South Sudan, and Kenya – only Kenya reported having a current CPD policy that links CPD requirements to periodic re-licensure.

During active practice, HCD participants noted that performance support is mainly limited to supervision visits and annual appraisals that aren't linked to learning efforts. They reported an increase in using digital solutions for supervision, such as tablets for data collection and virtual mentorship. Throughout the learning journey in the three deep-dive countries, participants consistently recommended providing mentorship for performance support, especially for new staff. A Kenyan participant emphasised the need for ‘effective mentorship for new staff’ and that ‘supportive supervision should include mentorship.’

### HW insights on preferred learning and performance interventions

Health workers ranked their preferences for learning approaches, digital platforms, and performance management strategies. Mentorship received the highest average score of 4.49 out of five, while eLearning alone had the lowest at 3.69 (**Table 1**). Six of the seven approaches scored above four, indicating strong agreement across all listed methods. The most preferred learning approaches were clinical rotations or practice (28.8%), clinical simulation-based training (18.6%), and blended learning (16.9%) (data not shown). There is some variation in preferences by country. Participants from Pakistan had the highest mean scores for point-of-care decision-making and mentorship and clinical rotations, indicating a preference for hands-on, workplace-based learning. Kenyan participants had the highest mean score for group-based problem-solving, which may indicate that quality improvement and a data-driven approach are preferred. South Sudan had the lowest mean preference score for all but three approaches, which may indicate a need for improved implementation or better adaptation of the approaches and content to the South Sudan context.

**Table 1:** Mean preference score for learning approaches by country (maximum score = 5)

	Country			
**Items**	**Kenya**	**Pakistan**	**South Sudan**	**Total sample**
I prefer to learn by				
*Mentorship – receiving workplace-based mentorship visits*	4.58	4.71	4.16	4.49
*Clinical rotations or clinical practice*	4.37	4.71	4.26	4.46
*Point-of-care decision-support mobile applications (for example, the US CDC sexually transmitted disease treatment mobile application)*	4.00	4.76	4.16	4.32
*In-service training – participating in group-based, instructor-led training*	4.16	4.52	4.05	4.25
*Group problem-solving – participating in workplace-based quality improvement activities (e.g. Plan-Do-Study-Act cycles, problem analysis, workflow re-organisation)*	4.74	4.43	3.53	4.24
*Peer-to-peer learning (in person)*	4.16	3.86	4.05	4.02
*Blended approach of in-person and digitally supported learning*	4.26	4.00	3.68	3.98
*Electronic learning – completing self-study courses*	4.05	3.24	3.84	3.69

US CDC – United States Centers for Disease Control and Prevention

When asked about preferred digital platforms for learning and performance, mobile social apps such as WhatsApp and Facebook topped the list, with 40.7% of participants ranking them first (**Table 2**). Additionally, HWs noted that older learners often resist digital learning, while younger users find it convenient and easy to adopt.

**Table 2:** Mean preference score for digital modalities (maximum score = 5)

	Country			
**Items**	**Kenya**	**Pakistan**	**South Sudan**	**Total Sample**
For digital modalities, I prefer using				
*Mobile social learning, such as WhatsApp groups, Facebook groups, etc.*	4.68	4.38	4.26	4.44
*Serious games – educational games on a digital device*	4.00	4.38	3.74	4.05
*Electronic learning – massive open online courses*	3.95	4.05	3.47	3.83

Participants preferred supervision from on-site supervisors or ward in-charge staff as a performance management intervention, with all options rated highly (maximum score out of five) (**Table 3**).

**Table 3:** Mean preference score for performance management approaches (maximum score = 5)

	Country			
**Items**	**Kenya**	**Pakistan**	**South Sudan**	**Total Sample**
For performance management, I prefer				
*Receiving supervision from the facility or ward in-charge*	4.37	4.19	4.47	4.34
*Receiving supportive supervision visits from the sub-national level*	4.42	4.29	4.16	4.29
*Participating in facility-based or results-based financing or incentives (e.g. funds delivered to the facility for specific outcomes)*	4.74	4.05	3.95	4.24

### HW insights on motivators

The HW HCD meetings identified several intrinsic and extrinsic motivators for improving performance. Common intrinsic motivations included the desire to perform to one’s best and to gain community recognition. A participant from Kenya said that there ‘should be recognition of excellent performance,’ and a participant from South Sudan noted that ‘non-rewarding of best performers leads to a lack of motivation.’ Additional opportunities to build capacity outside of their day-to-day job, including access to new digital solutions or platforms, information, and training, were mentioned as motivators. As noted by a participant in Kenya, ‘individuals doing well are motivated by being given a chance to attend seminars.’ In all three countries, advancing one’s career and increasing salary were major motivators for seeking learning opportunities, with anecdotal feedback indicating that per diems received when attending training were valued as supplements to low salaries.

Also mentioned were the importance of a conducive learning environment and an organisational culture that supports HW learning and performance. Participants from South Sudan noted a ‘bad working environment’ as demotivators, and participants from Kenya noted a ‘lack of complete records and clear action plans following supervision (visits) and performance evaluations’ as demotivators.

### National stakeholder insights on preferred learning and performance interventions

Country stakeholders reported that most funding for improving HW learning and performance is invested in in-service training and supportive supervision. When asked about other interventions they would like to increase, digital solutions were consistently identified as a priority. Government representatives reported favouring digital learning platforms that are non-proprietary, interoperable, affordable, and contextually appropriate.

Stakeholders expressed a desire to expand digital on-the-job training, viewing it as less expensive, possibly more effective, and likely to reduce absenteeism. Additionally, countries sought digital systems to improve data accessibility on HW performance and health outcomes, especially for supervision visits. They emphasised the need to link digital learning to human resource management and supervision systems in Kenya.

### National stakeholder insights on engagement with partners to improve HW learning and performance

EPI programs, in collaboration with national and international partners, manage the planning, delivery, monitoring, and evaluation of immunisation learning and performance interventions. Country stakeholders had mixed opinions on private-sector engagement, many expressed concerns about the private sector’s profit motives and noted a lack of experience with formal private-sector partnerships. Some believed that the private sector could contribute more to improving HW learning by providing opportunities for private-sector partners to engage more directly in national initiatives on HW learning and performance. Engagement levels with health professional associations and regulatory bodies varied widely: respondents from Pakistan and South Sudan reported no engagement, while those from Ghana and Kenya noted good relationships with these organisations but no direct coordination in national HW learning and performance efforts. In contrast, Burkina Faso participants mentioned active collaboration with professional associations in workshops and strategic decision-making.

### Common reported facilitators and barriers to adopting more effective and innovative interventions

The national stakeholders’ and HCD meetings identified common facilitators and barriers for expanding the use of both digital and non-digital interventions to improve HW learning and performance.

#### Protected time and staffing

HWs emphasised that providing protected time is essential for interventions requiring their participation during work hours, like on-the-job training and blended learning. One HW remarked, ‘unscheduled meetings after working hours cause demotivation,’ and added that adding protected time for on-site learning might be unrealistic. While HWs appreciate accessing digital learning online, they struggle to find time during the workday, as noted by a Kenyan participant who said clients do not like to see ‘you checking some information from your smartphone.’ Both national stakeholders and HWs identified heavy workloads and short staffing as barriers to participation in learning and performance interventions.

#### System and structural challenges

Structural barriers hinder countries from moving beyond classroom training and supervision. Stakeholders reported that training and supervision systems are mainly donor-funded and lack transparency regarding progress and outcomes. Factors such as the need to report on short-term deliverables, the simplicity of one-time training events, and financial incentives for training participation (*per diem*) can challenge the implementation of more complex interventions.

#### Adequate digital infrastructure and resources

Participants recognised that digital technology will increasingly support learning and performance interventions. However, national stakeholders cited barriers such as insufficient data on the effectiveness of digital practices, unclear policies on digital education integration, and the gender digital divide. Stringent laws for digital data collection and management can also hinder the uptake of digital interventions. Both HWs and national stakeholders identified sufficient digital infrastructure, mobile devices, and data access as critical for successful implementation. South Sudan HWs highlighted the cost of data and access to devices as barriers, with one saying, ‘Zoom and Teams use a lot of data (airtime), one person cannot afford daily.’ Many HWs mentioned digital literacy as a significant barrier, with a HW in Pakistan citing ‘negativity towards digitisation’ and a participant from South Sudan noting, ‘older people don’t see value in digital options.’

## DISCUSSION

Despite evidence of low effectiveness, stand-alone training and supervision remain priority investments for HW learning and performance and are often delivered in isolation. The analysis identified alternatives, such as quality improvement (also referred to as group problem-solving), blended learning, on-the-job training, and mentorship. While HWs favoured mentorship and stakeholders want to expand it, we did not find strong evidence of its effectiveness.

Barriers to expanding quality improvement and digital interventions include system and infrastructure challenges, short-term numbers-focussed reporting, the convenience of organising one-time events, and lack of digital infrastructure. According to the desk review, when a training for HWs does occur, it often takes the form of centralised, didactic trainings at district or capital levels. These trainings often prioritise senior and management staff who are incentivised with per diems received due to their participation in training. This marginalises and creates a disadvantage for rural health staff in a primary health care model that is mainly decentralised [14].

Country stakeholders have also reported that HWs used to *per diem* are often less motivated to attend and participate in other training interventions that do not provide financial reimbursement. The culture of per diems further encourages stand-alone training, making traditional interventions easier and more common, whereas more effective methods are more complex to implement.

Health professional associations and regulatory bodies help maintain quality in health professions, offering CPD opportunities in many countries. Nurses, the largest portion of the health workforce [15], are regulated in 86% of the countries included in the State of the World’s Nursing 2020 report [16]. CPD systems for nursing are in place in 68% of countries in WHO’s Africa region and 61% in the Southeast Asia region [16]. Completing CPD activities for licensure motivates ongoing learning, and HCD meeting participants highlighted CPD as a motivator. Yet, most learning and performance interventions operate separately from CPD policies; only one country mentioned such policies and stakeholders reported limited engagement with associations.

Continuing professional development activities linked to re-licensure are potentially sustainable, country-led processes that remain underutilised. However, CPD systems primarily benefit paid professional cadres represented by associations, such as doctors and nurses. Informal cadres such as CHWs lack similar representation and support. The capacity of regulatory bodies and professional associations varies significantly, and those in countries with newly established CPD policies may need capacity strengthening to implement them effectively.

Countries expressed a strong interest in expanding digital platforms to enhance HW learning and performance. These platforms provide continuous, accessible content, with HWs already using apps like WhatsApp and YouTube for quick information. However, the impact of digital interventions on learning outcomes remains mixed, and further evidence of their effectiveness and cost-efficiency is needed. Digital solutions are also crucial for performance management, with all countries prioritising digitised supervision data for real-time HW performance tracking. Across KIIs, the most common key facilitator for the scale-up of digital approaches was the involvement and commitment of the ministry of health to expand the use of digital tools. Some countries, like Burkina Faso, have demonstrated by setting up a department in charge of health information that supports digitalisation at all levels, including at the community level. Another key informant interviewer noted that health staff sometimes resist digital platforms, showcasing the social and behavioural barriers that affect the expansion of digitally supported learning and performance approaches. One country’s key informant noted that health staff are sometimes resistant to digital platforms. As technologies, such as artificial intelligence and decision-support systems grow [17], monitoring and evaluation will be essential to measure their impact.

Additionally, addressing gender and equity barriers is critical. Women, who comprise 70% of the health workforce [17,18], face digital access challenges; globally, women are 13% less likely to own smartphones and 15% less likely to access mobile internet, emphasising the need for more equitable access and digital literacy [19]. Closing this gap through building digital literacy and providing more equitable access to data and devices is vital.

Several strategies can promote learning and performance interventions beyond in-service training and supervision. First, it is crucial to educate national, donor, and implementing partner stakeholders on the effectiveness of these interventions. This education should shift the conversation from ‘training’ as the default solution to ‘training plus what else?’ Evidence exists that combining interventions enhances effectiveness. Donor funding guidance can encourage countries to adopt more effective interventions and require justification for relying on stand-alone, classroom-based training or supportive supervision.

Additionally, fostering actions that simplify the implementation of complex interventions is crucial. This can take many different forms, such as, but not limited to, capacity building on how to implement different interventions, *e.g.* providing guidance on work planning and budgeting for group problem-solving or multimodal interventions and strengthening digital infrastructure and literacy. In 2018, WHO provided guidance on implementing quality improvement that supports implementation [20]. Lastly, given stakeholder interest and the gap in evidence, it is important to advocate for evaluations of understudied interventions (*e.g.* certain digital interventions, mentorship) to fill gaps in the evidence base.

Country context and national priorities are crucial for decision-making. A country’s broader system for human resources for health, quality management, CPD maturity, digital infrastructure, and policies inform the selection of interventions to enhance HW learning and performance. Variability in infrastructure, geography, and health access means there is no ‘one-size-fits-all’ intervention. Thus, a needs assessment or situational analysis of current priorities and processes is essential for identifying relevant interventions that fit the country’s context.

### Limitations and strengths

This study had several limitations. First, only one KII was conducted in the four non-deep-dive countries due to time and resource constraints; ideally, we preferred to interview more EPI program managers and human resources for health in-service training representatives. Second, the desk review was limited to published systematic reviews and Gavi documents, without a systematic review of each intervention’s effectiveness. Third, there was no formal evidence grading during the desk review. Fourth, the situational analysis excluded CHWs, both because mid-level HWs deliver the majority of immunisation program services, and CPD systems, which influence HW motivation, do not apply to non-professional HWs. This makes the findings relevant to motivation, HW preferences, and contextual considerations non-generalisable to them. However, there are other analyses relevant to CHWs and digital learning that can guide CHW program managers [21]. Future assessments should focus on CHWs due to their significant role in health care.

Additionally, we did not explore in depth stakeholder perceptions of training and supervision effectiveness or HW motivation. HW reported preferences may have been influenced by prior exposure or the provided list of interventions. One strength of the study is the use of the HCD methodology, which encouraged collaboration and reflection among HWs and identified common barriers and facilitators, including digital solutions. Another strength is the inclusion of HW perspectives, addressing a gap in the literature.

## CONCLUSIONS

There are significant opportunities to expand HW learning and performance interventions that have proven effective, especially by combining them for greater impact. Key strategies include combining interventions, focusing on practice, moving beyond stand-alone training to ‘training plus what else?’ and linking training to quality improvement. Countries are interested in using digital platforms to provide quick access to performance data and more learning opportunities, but evidence of effectiveness is limited. Since HWs have limited access to learning opportunities, digital solutions can help. We recommend incorporating monitoring and evaluation into interventions that currently lack data on effectiveness.

## Additional material


Online Supplementary Document
