# Supplementary material for: Insights from national stakeholders and health workers on learning and performance interventions in immunisation programs: a multi-country situational analysis
Source: J Glob Health. 2025 Mar 28;15:04109. doi: 10.7189/jogh.15.04109 (PMC11952180; doi:10.7189/jogh.15.04109)
Supplement: Online Supplementary Document [file jogh-15-04109-s001.pdf]

**Supplement to: Bluestone J, Bryce E, Rowe AK, Ahuja N, Murathi WM, Njogu RN, Chandio A. Insights from national stakeholders and health workers on learning and performance interventions in immunisation programs: a multi-country situational analysis. J Glob Health. 2025;15:04109.**

TABLE S1. Supplementary Document. Learning and Performance Interventions Glossary of Terms

<table>
<tr><th>Term</th><th>Definition</th><th>Source of Definition</th></tr>
<tr><td>Clinical practice or clinical experiences</td><td>Generally, a preceptorship or observership and skill practice and learning with an expert, including observing and providing client care under supervision.</td><td>Billay D, Myrick F. Preceptorship: an integrative review of the literature. Nurse Educ Pract. 2008;8(4):258–66.</td></tr>
<tr><td>Simulation-based training</td><td>Practicing skills in a setting without real patients. Simulations are useful for building skills and self-efficacy for clinical scenarios that are rare, are unusually sensitive (e.g., counseling a suicidal child), or involve urgent life-and-death action (e.g., cardiopulmonary resuscitation).</td><td rowspan="2">Marinopoulos SS, Baumann MH, American College of Chest Physicians Health and Science Policy Committee: Methods and definition of terms: effectiveness of continuing medical education: American College of Chest Physicians Evidence-Based Educational</td></tr>
<tr><td>Point-of-care decision support</td><td>Information that is provided at the time of clinical need, integrated into a chart or</td></tr>
</table>

| Term | Definition | Source of Definition |
| --- | --- | --- |
| | electronic medical record, often accessible via mobile device or mobile application. | Guidelines. *Chest*. 2009;135(3 Suppl):17S-28S. |
| In-service training | Group-based training delivered by an instructor, in person or virtually; often delivered in a classroom, simulation center, or workplace. | |
| Educational outreach visits | On-the-job training strategy with face-to-face visits to individual HWs at their workplace by a person whom HWs regard as an expert or opinion leader to promote best practices. (May also be called "academic detailing.") | |
| Mentorship | A flexible teaching and learning process that serves specific objectives of the HW and health care service. This approach is relationship oriented. Mentorship is based on mutual trust and respect; it seeks to build confidence and is an empowering | Schwerdtle P, Morphet J, Hall H. A scoping review of mentorship in health personnel to improve the quality of health care in low and middle-income countries. *Global Health*. 2017; 13:77. |

| Term | Definition | Source of Definition |
| --- | --- | --- |
| | partnership between two people who have a shared set of learning objectives. | |
| Self-study, asynchronous training | On-the-job training with structured sessions in which HWs study independently without direct supervision or attendance in a class. Often includes content that can be accessed anytime (asynchronous), independent of the structured sessions. HWs might occasionally interact with a facilitator, supervisor, or peer to discuss the training content. | Adapted from: Rowe AK, Rowe SY, Peters DH, et al. The effectiveness of training strategies to improve healthcare provider practices in low-income and middle-income countries. *BMJ Global Health*. 2021;6:e003229. |
| Peer-to-peer training or learning | On-the-job training led by HW peers. For example, a HW (e.g., a facility in-charge) attends a training course, returns to their clinic, and shares the training information with other HWs. Also known as peer education. This strategy is different from educational outreach visits because peer-to-peer training does not involve visits by an external expert or opinion leader. | |

| Term | Definition | Source of Definition |
|---|---|---|
| Low-dose, high-frequency training | Short, simulation-based team learning at the job site, followed by deliberate, repeated peer-assisted practice activities. | Evans CL, Bazant E, Atukunda I, et al. Peer-assisted learning after onsite, low-dose, high-frequency training and practice on simulators to prevent and treat postpartum hemorrhage and neonatal asphysia. A pragmatic trial in 12 districts in Uganda. *PLoS ONE. 2018;* 13(2): e0207909. |
| Group problem-solving | Collaborative quality improvement or group problem-solving with or without formal teams. | Adapted from: Rowe AK, Rowe SY, Peters DH, et al. The effectiveness of training strategies to improve healthcare provider practices in low-income and middle-income countries. *BMJ Glob Health.* 2021;6:e003229. |
| HW-directed financial incentives | For example, performance-based payments linked to targets or client outcomes. | |

<table>
<tr><th>Term</th><th>Definition</th><th>Source of Definition</th></tr>
<tr><td>Supportive supervision</td><td>Improving routine supervision, benchmarking, or audit with feedback.</td><td rowspan="2">Handbook for national quality policy and strategy, a practical approach for developing policy and strategy to improve quality of care. Geneva: World Health Organization; 2018.</td></tr>
<tr><td>Quality improvement</td><td>An organizational strategy that formally involves the analysis of process and outcomes data and the application of systematic efforts to improve performance (also referred to as group problem-solving).</td></tr>
</table>

TABLE S2. Online Supplementary Document: Digital Education Interventions Glossary of Terms

| Term | Definition | Source of Definition |
| --- | --- | --- |
| Blended learning | The act of teaching and learning that integrates aspects of traditional and digital education. Blended education or learning can take diverse formats, depending on the type and the share of digital and traditional education employed in the blended educational approach. The digital component of blended learning includes online digital | *Digital education for building health workforce capacity. Geneva*: Geneva: World Health Organization; 2020. |

| | | |
|---|---|---|
| | education and the use of other digital education modalities. | |
| Digital education | Online or offline delivery of learning (often referred to as eLearning). | |
| Digital psychomotor skills trainers | A digital intervention to provide training in procedural skills that includes both manual tasks and clinical decisions. | |
| Serious gaming and gamification | A competitive activity in which students are given educational goals intended to promote knowledge acquisition. The games may be designed to promote learning or the development of cognitive skills, or they may take the form of simulations allowing learners to practice their skills in a virtual environment. | |
| Massive open online course | An online course that is designed for the participation of large numbers of geographically dispersed students (also referred to as eLearning or digital education). | |

| | | |
|---|---|---|
| Virtual reality | A technology that allows the user to explore and manipulate computer-generated real or artificial three-dimensional (3D) multimedia sensory environments in real time. It allows for a first-person active learning experience through different levels of immersion, i.e., a perception of the digital world as real and the ability to interact with objects and/or perform a series of actions in this digital world. | |
| Virtual patient | Interactive computer simulations of real-life clinical scenarios for medical training, education, or assessment. | |
| Mobile learning (m-learning) | Learning across multiple contexts, through social and content interactions, using portable, networked devices. | |
| Mobile-social learning | Incorporates use of mobile technology to increase access to digital learning opportunities and social platforms that encourage the social aspect of learning by | Guillaume D, Troncoso E, Duroseau B, Bluestone J, Fullerton J. Mobile-social learning for continuing professional |

| | | |
|---|---|---|
| | facilitating professional networks for sharing experiences and exchanging knowledge through virtual communication. | development in low- and middle-income countries: An integrative review. *JMIR Med Educ*. 2022;8(2):e32614. |
